# A Multimodal Imaging Approach Demonstrates Reduced Midbrain Functional Network Connectivity Is Associated With Freezing of Gait in Parkinson's Disease

**DOI:** 10.3389/fneur.2021.583593

**Published:** 2021-04-30

**Authors:** Amgad Droby, Elisa Pelosin, Martina Putzolu, Giulia Bommarito, Roberta Marchese, Luca Mazzella, Laura Avanzino, Matilde Inglese

**Affiliations:** ^1^Department of Neurology, Icahn School of Medicine at Mount Sinai, New York, NY, United States; ^2^Department of Neuroscience, Rehabilitation, Ophthalmology, Genetics and Maternal Child Health, University of Genoa, Genoa, Italy; ^3^Ospedale Policlinico San Martino, Istituti di Ricovero e Cura a Carattere Scientifico (IRCCS), Genoa, Italy; ^4^Asl3, Genovese Centri per i Disturbi Cognitivi e le Demenze (CDCD) Ponente, Genoa, Italy; ^5^Department of Experimental Medicine, Section of Human Physiology, University of Genoa, Genoa, Italy

**Keywords:** freezing of gait, functional connectivity, resting-state fMRI, Parkinson's disease, gait analysis

## Abstract

**Background:** The pathophysiological mechanisms underlying freezing of gait (FOG) are poorly defined. MRI studies in FOG showed a distinct pattern of cortical atrophy and decreased functional connectivity (FC) within motor and cognitive networks. Furthermore, reduced rs-FC within midbrain, frontal, and temporal areas has been also described. This study investigated the patterns of whole-brain FC alterations within midbrain inter-connected regions in PD-FOG patients, and whether these patterns are linked to midbrain structural damage using a multi-modal imaging approach, combing structural and functional imaging techniques.

**Methods:** Thirty three PD patients (16 PD-FOG, 17 PD noFOG), and 21 sex- and age-matched healthy controls (HCs) were prospectively enrolled. All subjects underwent MRI scan at 1.5T, whereas only PD patients underwent clinical and cognitive assessment. Grey matter (GM) integrity was measured using voxel-based morphometry (VBM). VBM findings served as basis to localize midbrain damage, and were further used as a seed region for investigating whole-brain FC alterations using rs-fMRI.

**Results:** In rs-fMRI, patients with PD and FOG demonstrated significant decrease of midbrain-cortical FC levels in the R PCG, right postcentral, and supramarginal gyri compared to controls and the middle cingulate compared to noFOG group. Based on the regression analysis, MOCA, UPDRS-III total score, and FOG severity scores were associated with FC levels in several frontal, parietal and temporal regions.

**Discussion:** The present results suggest that midbrain structural damage as well as decreased FC within the brainstem functional network might contribute to FOG occurrence in PD patients.

## Introduction

Freezing of gait (FOG), defined as a “brief, episodic absence or marked reduction of forward progression of the feet despite the intention to walk” (1), is a debilitating feature in Parkinson's disease (PD) (2) which have a dramatic impact on quality of life. FOG can be triggered or exacerbated by specific situations (e.g., turning, narrow passages) or can occur in response to increased motor (e.g., obstacle crossing), cognitive (dual task) and limbic (e.g., emotions) load. Indeed, besides motor symptoms, FOG can be associated with executive functions deficits, as well as mood disorders (e.g., anxiety) (1). The underlying pathophysiological mechanisms of FOG remain poorly understood. However, evidence suggests that (i) FOG does not originate from a single isolated brain region, but results from impaired communication within the corti co-subcortical-cortical circuitry and, (ii) the mesencephalic loco motor region (MLR) plays a role in this impaired communication. Among the different proposed models regarding FOG pathophysiology, the interference model by Nieuwboer and Giladi (3) suggests that FOG is a net result of top-down processing impairment, where breakdown of communication between motor, associative, and limbic pathways at a subcortical level leads to interruption of gait (4–6). Based on this, the interaction between these multiple failing neural circuits may underlie the heterogeneity of FOG features (7).

Structural MRI imaging studies have demonstrated patterns of gray matter (GM) atrophy including the left cuneus, precuneus, lingual gyrus, and posterior cingulate cortex in PD patients with FOG compared with PD patients without FOG and healthy individuals, and this GM decrease was correlated with performance in executive functioning (8). In white matter (WM), PD patients with FOG demonstrated reduced connectivity in the MLR involving mainly fibers connecting the peduncolopontine nucleus (PPN) with the medial frontal cortices and cerebellum (9–11).

Resting-state functional MRI (rs-fMRI) allows the investigation of the underlying pathophysiology in FOG on whole-brain functional network level (12, 13). Several rs-fMRI studies have reported reduced functional connectivity (FC) within several brain resting-state networks (RSNs) (14) including the sensorimotor network (SMN), the default mode network (DMN) (15) and the executive control network in PD patients with FOG (15–17). A study by Tessitore et al., reported altered FC within regions of the executive network and the visual functional network in PD with FOG, that was also associated with executive and visuo spatial performance (16). Furthermore, reduced rs-FC of the MLR and the cerebellum loco motor region with the supplementary motor area has been also described in PD patients with FOG, possibly reflecting a less automatic control of movement (17). Finally, rs-FC of the PPN, a major nucleus of the MLR, was also assessed, reporting an altered FC with visual temporal areas, as well as within the cortico-pontine-cerebellar pathways (18).

On these premises, we sought to investigate the patterns of whole-brain FC alterations within midbrain inter-connected regions in PD-FOG patients, and whether these patterns are linked to midbrain structural damage using a multi-modal imaging approach.

## Methods

### Participants

Fifty-four subjects (33 patients and 21 healthy controls) were prospectively enrolled in the study Patients with PD were classified as having FOG or not based on the score of the new FOG Questionnaire (FOG-Q) (19). Sixteen out of 33 patients who experienced FOG at least once a month (minimum score of 1 on item 2), for at least 2 sec (minimum score of 2 on item 4) were included in the PD-FOG group (5 female, mean age 72.43 ± 3.94 years), and 17 PD subjects (7 females, mean age 69.00 ± 5.43 years) were included in the PD-noFOG group. Twenty-one healthy subjects (HCs) (12 females, mean age 69 ± 13.5 years) served as a control group.

The inclusion criteria for patients with PD were: (a) age ≥ 18 years, (b) idiopathic PD according to the United Kingdom Parkinson's Disease Society Brain Bank criteria (20); (c) Hoehn and Yahr (H&Y) stage score ≤ 3, (d) stable PD pharmacological treatment for at least 1 month prior to the study; (e) ability to walk without any assistance for 2 min. Exclusion criteria for all participants were: (a) pre-existing history of neurological disorders other than PD, (b) psychiatric co-morbidity and history of psychiatric pharmacological treatments, (c) contraindications to Magnetic Resonance Imaging (MRI), (d) excessive head motion during MRI scan (>3mm average frame-wise displacement).

In PD patients, motor impairment was assessed with the Unified Parkinson's disease rating scale part III (MDS UPDRS-III) (20). PD participants were tested in the ON phase of their medication cycle (about 45 min after having taking anti-parkinsonian medications) both during MRI exam and during the clinical evaluation. Furthermore, in all participants global cognitive function was tested by using the Montreal Cognitive Assessment (MOCA) (21). All participants provided written informed consent prior to starting any procedures and after receiving an extensive explanation of the experimental protocol. This study was approved by the Ethics committee of the University of Genoa and each participant provided written informed consent prior to starting any procedures.

### MRI Data Acquisition

MR data were acquired for all patients using a 1.5 T Signa Excite (Signa Excite General Electric Healthcare, WI, USA) MR scanner with 8-channels phased-array head coil. The MRI protocol included: (a) high resolution Fast Spoiled Gradient Echo (FSPGR) 3-D T1-weighted sequence (TR/TE/TI = 11.56/5.048/500ms; FA = 8°, voxel size = 1 × 1 × 1 mm^3^), (b) axial T2-weighted (TR/TE1/TE2 = 2340/102/38.25ms; FA = 90°; voxel size = 0.94 × 0.94 × 4 mm^3^), and (c) 180 volumes resting-state fMRI datasets using single-shot echo-planar imaging (EPI) sequence (TR/TE = 3,000/60ms; FA = 90°, slice spacing = 1 mm, voxel size = 3.75 × 3.75 × 4 mm^3^).

### MRI Data Processing

Using a multi-modal imaging approach, we first investigated atrophy patterns of midbrain and cortex in PD-FOG relative to PD patients without FOG and HCs using voxel-based morphometry [(VBM); see Supplementary Material for further details]. VBM findings served as basis to localize midbrain damage, and further used as a seed region for investigating whole-brain FC alterations using rs-fMRI.

rs-fMRI datasets were pre-processed using SPM12 (https://www.fil.ion.ucl.ac.uk/spm/), where following steps were applied: slice time correction, re-alignment to the first volume image in order to correct for head movement, normalization to MNI using the parameters obtained from the segmentation procedure, and smoothing using an 8 mm full width at half maximum (FWHM) kernel. The de-noising and filtering of pre-processed rs-fMRI data sets were performed using the CONN (v.17) toolbox (McGovern Institute for Brain Research, Massachusetts Institute of Technology, Cambridge; https://web.conn-toolbox.org/), where, linear de-trending, temporal de-spiking, band-pass filtering (0.08 Hz – 0.1 Hz) were carried out after regression. The subjects' six head motion parameters, average WM, and CSF time course were entered as covariates of no interest in the regression model. Here, for each subject, functional connectivity (FC) maps of the brainstem network were calculated following seed-based approach, where a midbrain anatomical seed was applied based on the Harvard-Oxford Atlas available in CONN (see Supplementary Figure 1). Finally, the spatial accuracy of the constructed midbrain FC maps was visually inspected at both group–as well as individual level (22, 23).

### Statistical Analysis

All statistical analyses were performed using Statistical Package for Social Science (SPSS® v.23, IBM). Gender differences among the groups (PD-FOG, PD-noFOG and HC) were assessed by Chi-square test. Groups' difference at baseline for age and MOCA scores were assessed by the non-parametric Kruskal-Wallis test because data were not normally distributed. Two-sample *t*-test was used to detect differences between PD-FOG and PD-noFOG group for Hoehn and Yahr (H&Y) stage, for MDS-UPDRS part III total score and its gait sub-score (item 3.10). *P*-values < 0.05 were considered as a threshold for statistical significance.

Second level analysis of the MRI data was performed using SPM. 1 × 3 ANOVA was also conducted to assess between groups differences in FC within the brainstem functional network. The following contrasts were of interest: HC > FOG, noFOG > FOG, and HC > noFOG. Age, gender, and H&Y stage were entered as covariates in the design metrics of all the performed second-level analyses. Significance threshold for all between-groups statistical comparisons of interest was set to *p* < 0.001, family-wise error corrected for cluster size *(FWEc)* based on multiple-comparisons corrected using the Monte Carlo simulation (as implemented in Alphasim, AFNI).

Finally, a voxel-wise multiple regression was performed to explore the association between FC levels of brain regions within the midbrain network, disease severity (UPDRS III total score), gait performance (UPDRS III gait sub-score, item 3.10), cognitive performance (total MOCA score) in both patients' subgroups, and FOG severity (FOG-Q score) in PD-FOG only. Age, gender, and H&Y stage were included as covariates into the regression model. Significance threshold for all contrasts of interest was set to *p* < 0.01 and *FWEc* > 200 voxels.

Peak voxels β-weights within the detected clusters from the VBM, rs-fMRI, and regression analyses were extracted for further correlation analyses with clinical and performance scores.

## Results

### Participants' Characteristics

Of the total 54 participants enrolled to the study, 1 HC and 2 PD-noFOG were excluded due to motion artifacts, image artifacts, or low-quality images.

Detailed characteristics of the included participants can be viewed in Table 1. Briefly, no differences were observed in age and gender among the three groups (PD-FOG, PD-noFOG and HCs). As expected, patients with FOG had more severe motor impairments (*p* < 0.001 for UPDRS III and total score), higher H&Y score (*p* < 0.001) and worse cognitive performance compared with no freezers (*p* = 0.001).

**Table 1:** Demographic, and clinical characteristics of the study groups.

	**FOG**	**noFOG**	**HC**	
Gender (F/M)	5/11	5/10	11/9	*p* = 0.27
Age	72 ± 3	68.2 ± 5	64 ± 13.5	*p* = 0.056
H&Y stage	2.46 (2,3)	1.75 (1,2)	–	*p* < 0.001
Total UPDRS score	56.17 ± 24.33	32.93 ± 14	–	*p* < 0.001
UPDRS-III score	27.91 ± 16.27	18.87 ± 9.41		*P* < 0.001
UPDRS-III gait sub-score (item 3.10)	1.81 ± 1.10	1 ± 0.73		*p* = 0.02
FOG-Q	16.44 ± 8.9	–	–	–
MOCA-Total	23.4 ± 3.01	25.81 ± 2.79	–	*p* = 0.001
MOCA-Visuospatial	3.5 ± 1.9	4.4 ± 0.9	–	*p* = 0.001
MOCA-Attention	4.7 ± 1.2	5.7 ± 0.5	–	*p* = 0.001

*Values are reported as mean ± SD; H&Y data are expressed as median and range. H&Y, Hoehn & Yahr; UPDRS, unified Parkinson's disease rating scale; FOG-Q, freezing of gait questionnaire; MOCA, Montreal cognitive assessment*.

### Resting-State fMRI-Brainstem Network Functional Connectivity

**Figure 1:**
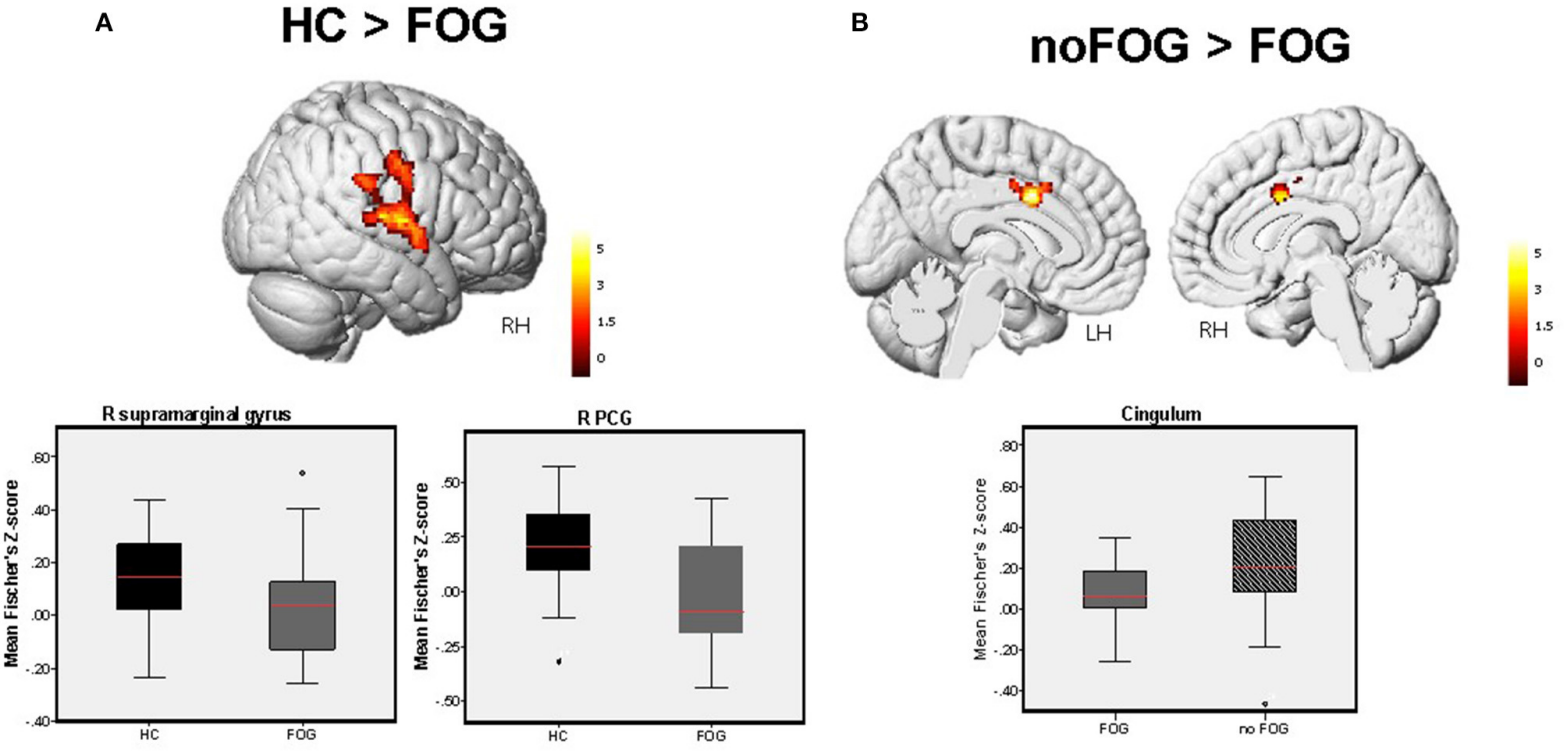
Between-group difference in FC levels within the brainstem network detected by one-way ANOVA (1x3) adjusted for age. **(A)** Significant FC decrease in the R PCG extending to the right supramarginal gyrus, and R STG was observed in PD-FOG vs. HCs. **(B)** Significant FC reduction in the cingulum was observed in PD-FOG compared to PD-noFOG. Bar charts demonstrate the mean and standard error of FC levels within these significant clusters across the three study groups. Results are reported at *p* < 0.001, *FWEc* in all cases.

Based on the calculated FC maps, frontal, parietal, and limbic brain regions were found to be functionally interconnected within the midbrain functional network (see Supplementary Figure 1). Based on the between-group comparisons of the calculated FC maps, PD-FOG patients showed significant FC decrease in the parieto-temporal cortex (right post central gyrus, right supra marginal gyrus, right superior temporal gyrus) compared to HCs (Figure 1A). Additionally, compared with PD-noFOG, PD-FOG patients exhibited decreased functional connectivity in the mid-cingulate (*p*_*FWEc*_ < 0.001) (Figure 1B). No significant differences were identified between PD noFOG patients and HCs. Results for functional connectivity within the brainstem network in the three groups are reported in Table 2.

**Table 2:** Brain regions showing between-group significant differences in FC within the brainstem network.

**Comparison**	**Brain region**	**MNI coordinates *x y z***	**Cluster size (# of voxels)**	***Z*-value**	***T*-value**	***p*-value**
HC > FOG	R PCG	64 −10 10	1,034	4.51	5.09	< 0.001^**^
	R supramarginal gyrus	64 −30 30				
	R STG	52 0 4				
noFOG > FOG	Middle cingulate	−6 4 36	261	4.61	5.23	0.025^*^

*RS, resting-state; FC, functional connectivity; MNI, montreal neurological institute; HC, healthy control; PD, Parkison's disease; FOG, freezing of gait; R, right; L, left; PCG, post- central gyrus; STG, superior temporal gyrus*.

* *p < 0.001;FWE_C_ cluster size > 450*,

** *p < 0.001; FWE_C_ cluster size > 200 voxels*.

### Voxel-Wise Multiple Regression Analysis

#### Disease Severity and Gait Performance

**Figure 2:**
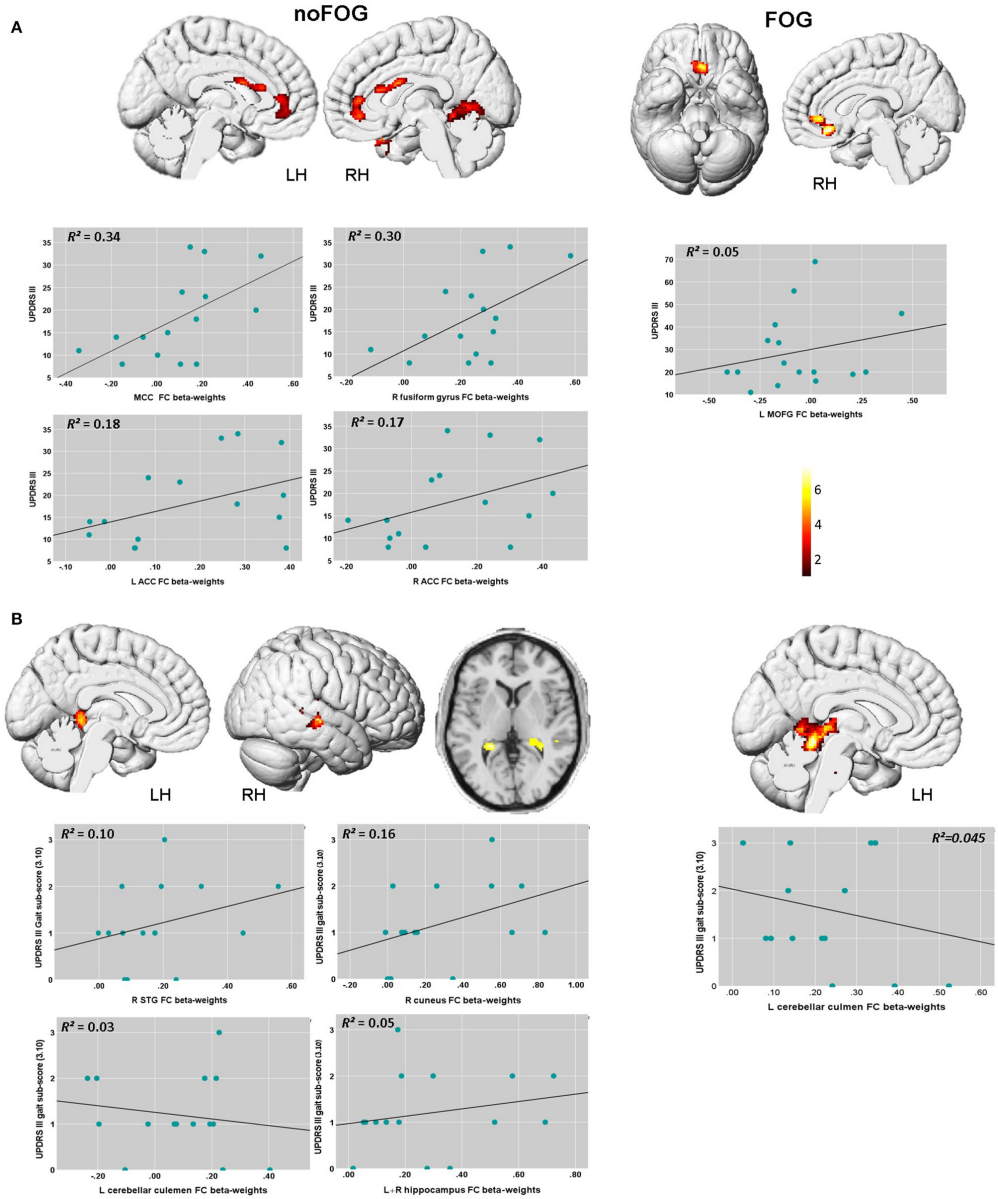
Brain regions within the brainstem network showing significant associations between FC levels, UPDRS III total score, and UPDRS-III gait sub-score (item 3.10) based on the voxel-wise regression analysis. **(A)** In the PD-NOFOG group, positive association between UPDRS III total score and FC levels were detected, with higher UPDRS III score associated with higher FC levels in the MCC, the right fusiform gyrus, and bilateral ACC. In PD-FOG group, UPDRS III total score correlated positively with FC levels in theL MOFG, and the right ACC. **(B)** In the PD-NOFOG group, association between gait sub-score in the UPDRS-III and FC levels were detected, with higher UPDRS III gait sub-score associated with increased FC levels in R STG, R cuneus, L hippocampus and lower FC levels in L cerebellar culmen. In the PD-FOG group, higher UPDRS III gait sub-score was associated with lower FC levels in L cerebellar culmen. Results are reported at threshold *p* < 0.01- FWEc, scatter plots depict the association between UPDRS-3 scores and FC β-weights in these clusters.

Regression analysis showed significant correlations between disease severity, gait performance and functional connectivity levels at threshold *p*_*FWEc*_ < 0.01 in both PD groups. The scatter plots in Figure 2 illustrate the association between the measured FC levels in these detected clusters, UPDRS-III total score and UPDRS-III gait sub-score (3.10).

UPDRS-III total scores were found to be significantly associated with FC levels in the left medial orbital gyrus (L MOFG), and the right anterior cingulate (ACC) in PD-FOG group (Table 3 and Figure 2A). In the PD-noFOG group, higher UPDRS-III total score was associated with increased FC levels in mid-cingulate cortex (MCC), the right fusiform gyrus, and left and right ACC (Table 3 and Figure 2A).

**Table 3:** Brain regions within the brainstem network showing significant correlations between UPDRS III total score and UPDRS III gait sub-score (item 3.10) and RS-FC levels based on voxel-wise multiple regression.

		**Brain region**	**MNI coordinates X Y Z**	**Cluster size (# of voxels)**	***Z*-value**	***T*-value**	*p-*value^*^
UPDRS III	*noFOG*	MCC	2 4 22	210	4.34	7.01	0.055
		R Fusiform gyrus	36 −64 −12	557	3.44	4.63	0.004
		L ACC	−4 40 6	293	3.13	4	0.027
		R ACC cingulate	6 42 6				
	*FOG*	L MOFG	−4 28 −14	298	3.39	4.42	0.024
		R anterior cingulate	6 38 −2				
UPDRS III gait sub-score (3.10)	*noFOG*	R STG	48 −22 −4	214	3.98	6.19	0.05
		R cuneus	22 −36 −2	452	3.39	4.66	0.007
		L hippocampus	−28 −38 −6	252	3.37	4.61	0.036
		L cerebellar culmen	−16 42 −16	232	3.11	4.07	0.043
	*FOG*	L cerebellar culmen	−28 −30 −34	1,373	4.54	7.7	0.001

*RS, resting-state; FC, functional connectivity; MNI, Montreal neurological institute; FOG, freezing of gait, R, right; L, left; MCC, mid-cingulate cortex; ACC, anterior cingulate cortex; MOFG, medial orbitofrontal gyrus; STG, superior temporal gyru;*

* *FWEc>200 voxels*.

In the PD-FOG group, worse gait performance (i.e., higher gait sub-score in the UPDRS-III) correlated negatively with FC levels in the left cerebellar culmen (Table 3 and Figure 2B). UPDRS-III gait sub-scores were positively associated with FC in the right superior temporal gyrus (R STG), right cuneus, and left hippocampus, and negatively in the left cerebellar culmen (Table 3 and Figure 2B)

**Figure 3:**
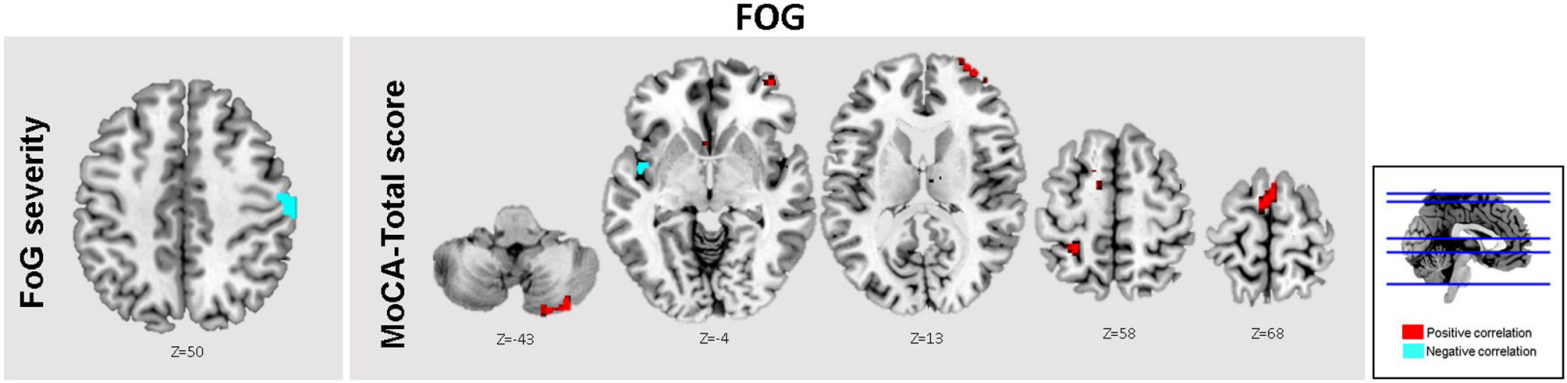
Brain regions within the brainstem network showing significant associations between FC levels, and clinical scores. In the FOG group, the total MOCA scores were significantly associated with FC levels in the A ACC, SMA, L MFG, and bilateral cerebellar crus II (Red). Moreover, significant inverse associations between FOG severity scores and FC levels within the R PCG (Cyan). Results are reported at threshold *p* < 0.01-*FWE*c.

Finally, in PD-FOG, severity of freezing (i.e., FOG-Q score) correlated negatively with FC levels within the right post central gyrus (R PCG) (Table 4 and Figure 3).

**Table 4:** Brain regions within the brainstem network showing significant correlations between performance in MOCA, FOG severity, and RS-FC levels based on voxel-wise multiple regression.

		**Brain region**	**MNI coordinates X Y Z**	**Cluster size (# of voxels)**	***Z*-value**	***T*-value**	***p-*value**
**MOCA-Total**								
	*FOG*	L Anterior cingulate	−16 30 22	4,115	4.68	8.71	<0.001^*^
		L MFG	−30 30 32		4.58	8.28	
		SMA	2 8 72	1,870	4.58	8.28	<0.001^*^
		L PCG	−20 −46 36				
		R cerebellum (Crus II)	24 −87 −50	702	3.85	5.82	0.001^*^
**FOG severity**								
	*FOG*	R PCG	56 −6 48	218	3.48	4.86	0.032

*RS, resting-state; FC, functional connectivity; MNI, Montreal neurological institute; MOCA, Montreal cognitive assessment battery for dementia; PD, Parkison's disease; FOG, freezing of gait; R, right; L, left; MFG, middle frontal gyrus; SMA, supplementary motor area; PCG, post central gyrus;*

* *FWEc>200 voxels*.

#### MOCA

Based on the correlation analysis between MOCA scores and the extracted FC β-weights from the detected clusters in the regression analysis at threshold *p*_*FWEc*_ < 0.01, significant correlations between FC levels and total MOCA scores were obtained in the L ACC (*r*^2^ = 0.39), the right cerebellar crus II (*r*^2^ = 0.05), and SMA (*r*^2^ = 0.11) (Table 4 and Figure 3). No significant clusters were found to be associated with the MOCA scores in the noFOG group.

## Discussion

Here we applied a multimodal neuroimaging approach combing structural and functional imaging techniques for the study of whole-brain neural correlates of midbrain damage in age-and-gender matched PD-FOG and PD-noFOG patients. In a multi-step approach, we first identified GM atrophy using VBM, midbrain regions showing a significant GM decrease in PD with FOG compared to PD without FOG and healthy controls from the VBM analysis were used as seed region for midbrain FC calculation.

As a first result, patients with PD and FOG exhibited a greater GM atrophy in midbrain structures, as well as in parietal regions (including the bilateral precuneus and cuneal regions), thalamus and putamen compared to PD-FOG.

The observed patterns of cortical GM atrophy are in accordance with those reported by previous studies, in which significant difference in frontal and parietal GM volumes in FOG patients with respect to healthy and PD-no FOG participants (8, 24), where this pattern of GM atrophy was found to associated with FOG severity. Compared with PD-noFOG group, PD-FOG patients showed significant GM decrease in midbrain structures, thalamus, and putamen suggesting that damage in these subcortical regions might be linked to the development of FOG. GM decrease in the mesencephalic motor regions (MLR) and thalamus in freezers compared with non- freezers (22) was previously described, where thalamic atrophy in PD-FOG patients was found to be associated with visual recognition performance (25). These findings further highlight the role of these sub-cortical structures in the multi-sensory integration process where they play a key role in sustaining gait (7, 26). This is further supported by our obtained rs-fMRI results.

Based on the rs-fMRI analysis, here we found that patients with PD and FOG demonstrated significant decrease of midbrain-cortical FC levels in the R PCG, R supra marginal gyrus, and R STG compared to controls; and the middle cingulate compared to no-FOG group. Such patterns of FC alterations in FOG patients involving associative frontal, parietal, and limbic brain areas implies that higher cognitive networks that are involved in attention and visuo motor integration also give rise to gait disturbances in FOG (6, 27–29). Furthermore, the regression analysis with the UPDRS III total score, and UPDRS III gait performance sub-score showed that FC in a complex network involving the anterior and middle cingulate cortex, the fusiform gyrus and the superior temporal gyrus was associated with disease severity and worse gait performance in PD-noFO group, but not in PD patients with FOG. Differently, in both PD-FOG and PD-noFOG groups, FC connectivity of L cerebellar culmen was associated with better gait performance (lower scores at UPDRS III gait performance sub-score).

A possible explanation is that recruitment of compensatory areas that are involved in higher order functions, such as connecting emotion to motor control (30), multisensory integration (31), and integration for both egocentric and object-centered reference systems (32) might not be sufficient anymore for improving motor performance deficits in PD-noFOG group (maladaptive compensation), whereas the additional recruitment of cerebellum leads to better gait performance (adaptive compensation). In PD patients with FOG, we detected only adaptive compensatory activity of L cerebellar culmen in counteracting gait disturbances, possibly reflecting lower neural reserve.

Our rs-fMRI findings are all related to midbrain-cortical FC patterns. The pendunculopontine nucleus (PPN) is a major nucleus of the MLR, and has been implied in gait control and FOG pathophysiology [for a review see (33)]. Experimental data on neurophysiology of the PPN strongly support that PPN participates in higher functions of the brain. A considerable number of PPN cholinergic fibers innervate the centro median-para fascicular (CM-Pf) complex of the thalamus, which in turn innervates the cerebral cortex (34–36). Thus, the CM-Pf nucleus allows the PPN to gain access to different cortical functions and to several high-order functions of the brain, including attention, action selection, learning, memory, spatial perception, control of impulsivity and decision-making. PPN neurons may also participate in these functions through their axons ending in the striatum, where they synapse onto medium spiny neurons and cholinergic interneurons (37). In line with this, local field potentials recordings from PPN- implanted electrodes for deep brain stimulation in PD patients have demonstrated peaks in both alpha and beta bands, coherent with the cortex (38–41). Whilst beta band activity recorded from the basal ganglia and cortex has been linked to motor function, increasing evidence indicate that alpha oscillatory activity has an important role in attention and the allocation of processing resources (42). A magneto encephalography (MEG) study reported that these two PPN oscillatory bands show distinct connectivity patterns – with the beta band network coherent with the supplementary motor area (SMA), as well as the alpha network including the posterior mid-cingulate that is involved in integrating sensorimotor feedback during movement (43, 44). Our current findings further corroborate these reports, demonstrating that FOG, worse gait, as well as cognitive performance are associated with reduced midbrain-cortex FC in a large network including parieto-temporal cortex, and mid-cingulate.

Nonetheless, the limitations of the study deserve to be discussed. Firstly, our multimodal investigation was at 1.5T MR field strength, allowing a sub-optimal delineation of the PPN and other neighboring midbrain anatomical structures. Secondly, the relatively small sample size is also a limitation. However, it is worth pointing out that both PD groups were well-matched in terms of age and gender, but differed significantly in UPDRS and MOCA scores, which is expected as FOG is more commonly observed in the later stages of the disease (1). Moreover, PD patients enrolled to this were in a mid-stage of the disease, thus future studies should also include patients in an earlier and more advanced stage of the disease in order to better investigate the relationship between disease severity and local GM reduction in FOG.

In conclusion, using a multimodal approach, the present study offers novel information on the role of midbrain in FOG pathophysiology. Based on our results, we postulate that in the advanced stages of PD, structural integrity loss of the midbrain and the accompanying FC reductions between these structures and brains' higher cortical regions contribute to the occurrence and severity of FOG.

## Data Availability Statement

The raw data supporting the conclusions of this article will be made available by the authors, without undue reservation.

## Ethics Statement

The studies involving human participants were reviewed and approved by the ethics committee of the University of Genoa, Italy. The patients/participants provided their written informed consent to participate in this study.

## Author Contributions

AD and MI: data analysis, writing, and finalizing manuscript. EP and LA: data collection, writing, and finalizing manuscript. MP and GB: data collection and revising manuscript. RM and LM: revising manuscript. All authors contributed to the article and approved the submitted version.

## Conflict of Interest

EP: received research grants from Italian Ministry of Health and the Michael J. Fox foundation. LA: received research grants from Italian Ministry of Health and Gossweiler Foundation. MI: received research grants from NIH, DOD, NMSS, FISM, and Teva Neuroscience; received fees for participating in advisory boards from Roche, Biogen, Merck and Genzyme. The remaining authors declare that the research was conducted in the absence of any commercial or financial relationships that could be construed as a potential conflict of interest.
