# Supplementary material for: A Multimodal Imaging Approach Demonstrates Reduced Midbrain Functional Network Connectivity Is Associated With Freezing of Gait in Parkinson's Disease
Source: Front Neurol. 2021 Apr 30;12:583593. doi: 10.3389/fneur.2021.583593 (PMC8120105; doi:10.3389/fneur.2021.583593)
Supplement: Supplementary file 1 [file Data_Sheet_1.docx]

**Supplementary Material**

**Methods**

*Voxel-based morphometry (VBM) analysis*

VBM was performed using statistical parametric mapping (SPM12; https://www.fil.ion.ucl.ac.uk/spm/software/spm12/) and the diffeomorphic anatomic registration Exponentiated Lie Algebra (DARTEL). The T1-weighted images were segmented producing grey matter (GM), white matter (WM), and cerebrospinal fluid (CSF) probability maps. The tissue probability maps were normalized to MNI space and then smoothed using an 8 m”m FWHM kernel. Second level analysis of the MRI data was performed using SPM. 1×3 ANOVA was used to assess between groups differences in GM densities between the study groups. The following contrasts were of interest: HC > PD all, HC > FOG, noFOG > FOG, and HC > noFOG.

**Results**

*GM structural differences: Voxel-based morphometry (VBM)*

At threshold of p < 0.001, *FWEc* > 450 voxels), reduced GM densities were observed in a cluster extending from the left superior parietal gyrus to the left precuneus and in a cluster extending from right precuneus to the right cuneus were found in PD-FOG patients when compared with HCs. Compared to PD-noFOG, PD-FOG patients (**Supplementary Figure 2**) showed a significant reduction in grey matter volume in a cluster extending from the left thalamus to the midbrain and the right thalamus to the right putamen. No significant differences were detected between HC and noFOG groups (See **Supplementary** **Table 1**).

**Supplementary Table 1:** Brain regions showing between-group structural significant differences (VBM).

|  | **Comparison** | **Brain region** | | **MNI coordinates**  ***x y z*** | | **Cluster size**  **(# of voxels)** | ***Z*-value** | ***T*-Value** | ***p*-value** |
| --- | --- | --- | --- | --- | --- | --- | --- | --- | --- |
| ***Between-groups structural differences (VBM)^*^*** | HC > FOG | L SPG | | -14 -66 16 | 1176 | | 4.19 | 4.65 | < 0.001 |
|  |  | L precuneus | | -16 -74 50 |  | |  |  |  |
|  |  | R precuneus | | 10 -74 38 | 452 | | 4.06 | 4.48 | 0.008 |
|  |  | R cuneus | | 16 -66 14 |  | |  |  |  |
|  | noFOG > FOG | L thalamus | | -14 -6 8 | 1274 | | 5.67 | 6.87 | < 0.001 |
|  |  | Midbrain | | 6 -30 -8 |  | |  |  |  |
|  |  | R thalamus | | 12 -8 4 | 715 | | 5.41 | 6.43 | 0.001 |
|  |  | R putamen | | 20 -8 14 |  | |  |  |  |
|  |  |  | |  |  | |  |  |  |
|  |  |  |  | |  | |  |  |  |

RS= resting-state, FC = functional connectivity, MNI: Montreal Neurological Institute, HC = healthy control, PD = Parkison’s disease; FOG = freezing of gait, R = right, L = left, SPG = left superior parietal gyrs, PCG = post- central gyrus, STG = superior temporal gyrus. ^*^ = *p* < 0.001; *FWE_C_* cluster size > 450, ^**^ = *p* < 0.001; *FWE_C_* cluster size > 200 voxels

**Figure Legend**

**Supplementary Figure 1:** Depiction of the midbrain seed ROI (left), and the calculated midbrain network functional connectivity map.

**Supplementary Figure 2:** Voxel-based morphometry (VBM) to detect between-groups structural differences using one-way ANOVA (1x3) adjusted for age. (a) VBM analysis revealed a significant GM reduction in bilateral precuneus, R cuneus, and the L SPG in FOG vs. HCs. (b) In FOG vs. no-FOG, a reduction in the left and right thalamus, midbrain, and right putamen were detected. Results are reported at p < 0.001, *FWEc* in all cases.
