# Supplementary figures and images for: A Multimodal Imaging Approach Demonstrates Reduced Midbrain Functional Network Connectivity Is Associated With Freezing of Gait in Parkinson's Disease
Source: Front Neurol. 2021 Apr 30;12:583593. doi: 10.3389/fneur.2021.583593 (PMC8120105; doi:10.3389/fneur.2021.583593)

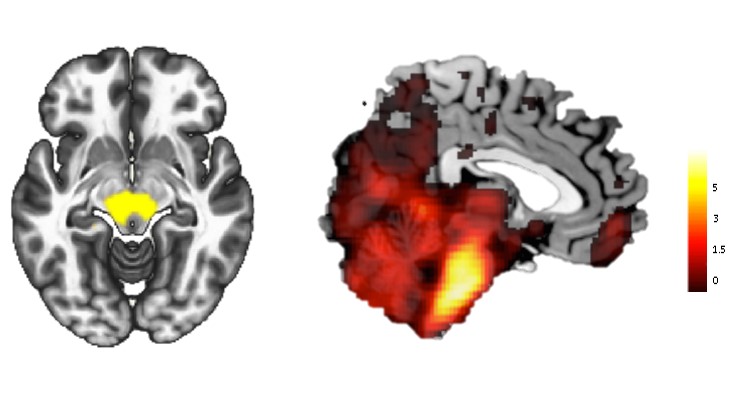

Supplement: Supplementary file 2 [file Image_1.jpeg]

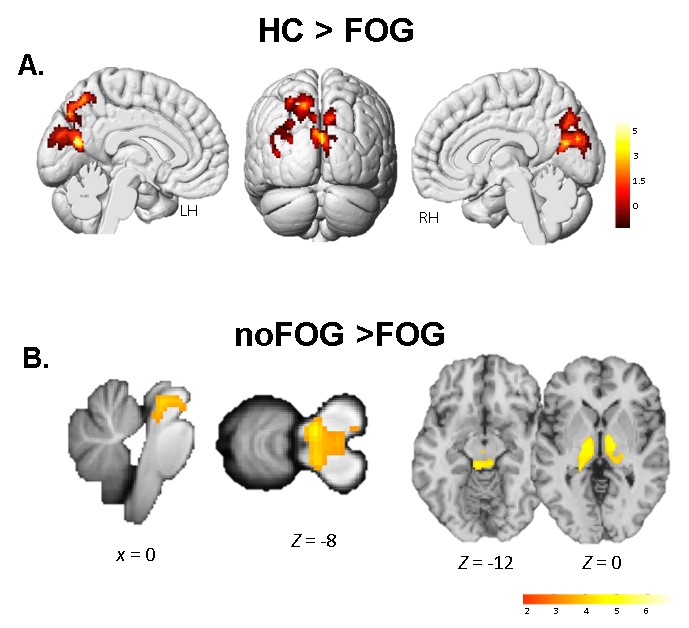

Supplement: Supplementary file 3 [file Image_2.jpeg]
